# Data-driven prediction of diamond-like infrared nonlinear optical crystals with targeting performances

**DOI:** 10.1038/s41598-020-60410-x

**Published:** 2020-02-26

**Authors:** Rui Wang, Fei Liang, Zheshuai Lin

**Affiliations:** 1 University of Chinese Academy of Sciences, Beijing, 100190 China; 2 Technical Institute of Physics and Chemistry, Chinese Academy of Sciences, Beijing, 100190 China

**Keywords:** Nonlinear optics, Computational methods

## Abstract

Combining high-throughput screening and machine learning models is a rapidly developed direction for the exploration of novel optoelectronic functional materials. Here, we employ random forests regression (RFR) model to investigate the second harmonic generation (SHG) coefficients of nonlinear optical crystals with distinct diamond-like (DL) structures. 61 DL structures in Inorganic Crystallographic Structure Database (ICSD) are selected, and four distinctive descriptors, including band gap, electronegativity, group volume and bond flexibility, are used to model and predict second-order nonlinearity. It is demonstrated that the RFR model has reached the first-principles calculation accuracy, and gives validated predictions for a variety of representative DL crystals. Additionally, this model shows promising applications to explore new crystal materials of quaternary DL system with superior mid-IR NLO performances. Two new potential NLO crystals, Li_2_CuPS_4_ with ultrawide bandgap and Cu_2_CdSnTe_4_ with giant SHG response, are identified by this model.

## Introduction

As a class of optoelectronic functional materials, nonlinear optical (NLO) crystals enable many important applied communities in laser frequency conversion, quantum information, optical communications and other fields^1–4^. Especially, in the mid-IR spectral range of 3**–**25 μm, as the fingerprint region of organic and inorganic molecules, the searching of good NLO crystals is in urgent demand to obtain coherent generation. To date, all commercial mid-IR NLO crystals are semiconductor chalcopyrites, such as AgGaS_2_, AgGaSe_2_ and ZnGeP_2_^5^_._ However, they suffer from some intrinsic shortcomings, e.g., low laser damage threshold (LDT)^6^, strong two-photon absorption^7^, and difficult/dangerous crystal growth^8^, which hinder their wide applications in high-power laser industry. Therefore, it is still a challenging task to promote and develop new mid-IR crystals with superior NLO performances.

Generally, a practical mid-IR NLO crystal should meet the following requirements^9^: (i) good IR transparency in the important mid-IR atmospheric window (3**–**5 μm and 8**–**12 μm); (ii) large SHG coefficient *d*_ij_ (at least larger than 10 × KDP (*d*_36_ ≈ 0.39 pm/V) and at best larger than AgGaS_2_ (*d*_36_ ≈ 13.4 pm/V)); (iii) high LDT. For a good mid-IR NLO crystal, the energy band gap *E*_g_ should be more than 3.0 eV; (iv) moderate birefringence Δn (∼0.03 − 0.10); (v) easy crystal growth and chemical stability. It should be noted that a critical problem for the development of mid-IR NLO crystals is to fulfill the suitable balance between the large SHG response (*d*_ij_) and enough band gap (*E*_g_) in the light of their inverse dependence. Though many researchers aimed at mid-IR crystals and involved structure-property relationship in recent years, the traditional trial-and-error experiments and first-principle simulations are still time-consuming and laborious. Therefore, first-principles prediction combining high-throughput screening and machine learning is a burning issue for rapid development of mid-IR NLO crystals.

With the introduction of “Material Genome Project”^10^, it has become a new research hotspot in the field of material science for combining High-Throughput Computing (HTC) with Machine Learning (ML) models to clarify the inherent structure-property relationship of materials and to accelerate the research and development of new materials, especially of functional materials, such as thermoelectric materials, low dimensional materials, solar cell, superconductors and superhard materials^11–18^. For example, in 2018, Brgoch *et al*. identified a new promising phosphor (NaBaB_9_O_15_) for solid state lighting via ML^19^. The further experiments verified this new phosphor with a high quantum yield (95%) and excellent thermal stability. In 2019, Braatz *et al*. accurately applied machine-learning tools to predict and classify lithium-ion battery cycle life before capacity degradation with test errors less than 10%, providing a promising route for prognostics and diagnostics of lithium-ion batteries^20^. This work also highlighted the prospects of data-driven modeling to predict the behavior of complex systems.

Herein, for the first time, we employ ML models into the area of NLO crystals, aiming to provide more insights for exploring new mid-IR NLO crystals fulfilling the good balance between *d*_*ij*_ and *E*_*g*_. In particular, the cubic close- packed diamond-like (DL) structures are selected to be the candidate dataset because of their promising NLO properties. The model’s predictions on the SHG coefficients of commercial NLO DL crystals are in good agreement with first-principles simulations. Remarkably, two superior NLO crystals, Li_2_CuPS_4_ with a wide forbidden gap and Cu_2_CdSnTe_4_ with giant nonlinearity, are predicted and wait for further experimental verifications.

## Dataset

In past few years, metal chalcogenides with DL structures have received increasing attention as they provide an attractive performance tuning dataset for a range of important applications, including lithium-ion conductors^21^, band gap adjustable semiconductors^22^, solar cells^23^, thermoelectric materials^24^ and nonlinear optics^25^. Several commercially NLO crystals belong to the DL structure. AgGaS_2_ exhibits a large SHG effect (~13 pm/V) and possesses a wide transparent window (from 0.74 to 13 μm); it also acts as a standard crystal to evaluate other crystals’ performance^26,27^. ZnGeP_2_ (ZGP) is the most important and widely used NLO crystal in 3–5 μm due to its multiple advantages^28^. CdGeAs_2_ owns the currently largest SHG coefficient (*d*_36_ = 236 pm/V) and has been successfully applied to the difference-frequency generation (DFG) and optical parametric generation (OPG)^6,29^. In 2017, Liang *et al*.^30^ systematically summarized and analyzed the structure and property relationships in mid-IR NLO metal chalcogenides, and proposed that polar aligned DL structure would be the most favorable system due to its large band gap, sufficient SHG effect, moderate birefringence, and good crystal growth habit and chemical stability. After then, quite a few experiments demonstrated this prediction, such as for Hg_2_GeSe_4_^31^, Li_4_HgGe_2_S_7_^32^, and Ga_2_Se_3_^33^. Therefore, the similar stacking type and abundant composition in the DL structure definitely enables the exploration for superior mid-IR crystals in the context of ML and HTC.

**Figure 1:**
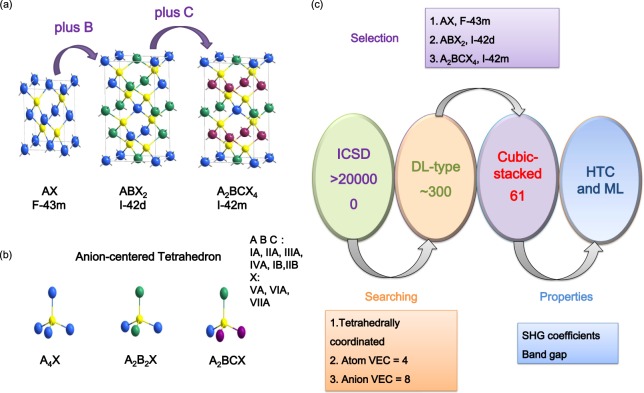
(**a**) Crystal structures of cubic-stacked DL compounds, AX, ABX_2_ and A_2_BCX_4_. (**b**) The anion-centered tetrahedron as the basic unit in DL structures. (**c**) The screening workflow for DL-type crystals stacked cubically.

In this paper, we mainly considered three classes of DL crystals, AX, ABX_2_ and A_2_BCX_4_ (A, B, C = IA, IIA, IIIA, IVA, IB, IIB cations; X = VA, VIA, VIIA anions) which belong to the space groups F-43m,I-42d and I-42m, respectively. As shown in Fig. 1a,b, the splitting of the cation site in AX leads to the ternary compound ABX_2_ and quaternary compound ABCX_4_, in which all of their basic structural units are tetrahedral. In these three kinds of crystal structures, all anion-centered tetrahedrons are arranged along the [111] direction, which is very beneficial for the superposition of microscopic second-order susceptibility of units. After a screening (Fig. 1c) in the Inorganic Crystallographic Structure Database (ICSD)^34^, totally 61 DL structures (26 binary compounds, 29 ternary compounds and 6 quaternary compounds) were collected. It should note that all these compounds, except the quaternary compound Li_2_CuPS_4_, was experimentally synthesized and their crystal structure data has been determined.

## First-Principles Methods

To provide the learning data for machine learning, the first-principles simulations were fulfilled by the plane-wave pseudopotential method^35^ based on density functional theory (DFT)^36^ implemented in the CASTEP module^37^. The cell parameters and atomic positions in the unit cells of all crystals were firstly optimized using the BFGS method^38^ with the convergence criterion of 5×10^–6^ eV/atom, 0.01 eV/Ǻ, 0.02 GPa, and 5.0 × 10^−4^ Ǻ for energy change, maximum force, maximum stress, and maximum displacement, respectively, between two consecutive processes. The exchange-correlation functionals were described by the local density approximation (LDA)^39^. A kinetic energy cutoff 880 eV and Monkhorst-Pack k-point meshes^40^ spanning less than 0.07 Ǻ^−3^ in the Brillouin zone were chosen. We used the screened-exchange local density approximation (sx-LDA)^41^ in the calculation of electronic structure in order to obtain the accurate band gap *E*_*g*_. The static SHG coefficients *d*_*ij*_ are calculated using an expression originally proposed by Rashkeev *et al*.^42^. It has been revealed from previous researches that our first-calculations provided a good estimate of NLO properties in IR sulfide crystals, as demonstrated in LiGaS_2_^43^, AgGaS_2_^44^, BaGa_4_S_7_^9^, and LiZnPS_4_^9^. The calculated values of band gaps *E*_*g*_ and SHG coefficients *d*_*ij*_ for the common DL NLO materials are listed in the supplementary information file.

## Machine Learning Methods

### Random forest regression

**Figure 2:**
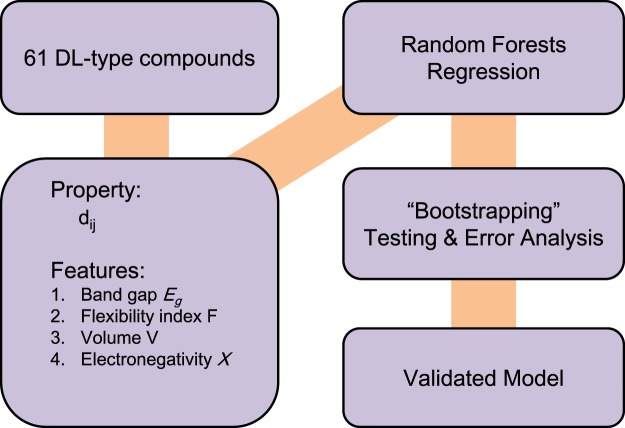
Overall workflow for the machine learning model. Four atomic or structural features are generated for the Random Forests Regression, in which “bootstrapping”^55^ and performance evaluation are used to validate the model, leading to a predictive model for the SHG coefficient of NLO crystals.

We propose to use Random Forests Regression (RFR) to predict the target variable, SHG coefficients in the selected NLO crystals (Fig. 2). For the RFR, the Scikit-learn package in Python was used^45^.

RF is an integrated algorithm that combines multiple decision trees, with the advantages of good generalization performance, insensitivity to data outliers and fewer hyper parameters. RFR’s training procedure was first proposed by Breiman^46^. (1) Acquire a random bootstrap sample from the data set. (2) For each bootstrap sample, nurture a tree with the following rule: at each node, find the best split point by a specific feature. The split criterion is to maximize the Information Gain (IG), which can be defined (for a binary split) as

1 $$IG({D}_{P},{x}_{i})=I({D}_{P})-\frac{{N}_{left}}{{N}_{p}}I({D}_{left})-\frac{{N}_{right}}{{N}_{p}}I({D}_{right})$$

*x*_*i*_ is the feature, *N*_*p*_, *N*_*left*_ and *N*_*right*_ are the number of sample at the parent node and two child nodes, respective, *I*_*p*_, *I*_*left*_ and *N*_*right*_ represent the impurity function for the parent and child nodes, respectively. The impurity index *I*(*t*) at the node *t* can be calculated as

2 $$I({D}_{t})=MSE(t)=\frac{1}{{N}_{t}}\sum _{i\,\in {D}_{t}\,}{({y}^{(i)}-{\hat{y}}_{t})}^{2}$$

*N*_*t*_ is the number of sample at *t* node, *y*^(*i*)^ is the true target value and ${\hat{y}}_{t}$ is the average target value of the sample.

The RFR model uses the mean squared error (MSE) criteria to nurture every decision tree and the average value of the decision trees predicts the target variable.

### Feature selection

The representations of a crystal, called “descriptors” or “features”, play an indispensable role in applying ML model to predict its physical properties. In materials science, descriptors can be divided into elemental or structural representations^47^. The selection of descriptor candidates is an essential part in constructing a ML model, which need not only satisfy the necessary specific requirements (e.g. dimensional invariance for chemical compositions), but also reflect physical meanings related to the target variable. Based on the existing knowledge, we chose four features to represent the DL crystals, *i.e*., band gap, flexibility index, ionic group’s volume and the electronegativity of anions.

(i) Energy band gap Eg. The band gap is a physical quantity closely related to the SHG coefficients. Generally, for the similar structure and composition, they have a negative correlation^48^. The band gap of a crystal can be acquired by first-principles simulations or experiments (e.g. Photoluminescence). Here, we choose the experimental value of the band gap as one of features.
(ii) Flexibility index F. In 2014, Jiang et al.^49^ proposed a flexible dipole model based on the concept of bond-valence. The results showed that the magnitude of NLO effects was determined by the compliance of the dipole moment in response to external disturbances. “Flexibility index” between two connected atoms is defined as
  3 $$F=\frac{{\exp }[({R}_{0}-{R}_{l})/C]}{{(\sqrt{{C}_{A}}+\sqrt{{C}_{B}})}^{2}/{{R}_{l}}^{2}}\times \frac{1}{({X}_{A}-{X}_{B})}$$
  where *R*_*l*_ is the distance of two atoms, *R*_0_ is the tabulated ideal bond length in the bond-valence theory, *C*_*A*_ (or *C*_*B*_) is the number of valence electrons of atom A (or B), *X*_*A*_ (or *X*_*B*_) is the electronegativity of atom A (or B) and *C* is an empirical constant, typically 0.37 Ǻ. For a tetrahedron, the *F* was calculated by averaging four values of the coordinated atoms.
(iii) Volume *V*. The optical properties of a crystal have closely relations with the volume and density of groups^50^. Here we only consider the volume of the ionic groups (tetrahedra) because all crystal structures are in closely packed configurations.
(iv) Pauling Electronegativity *PE*. As the scale of the ability of atoms to attract electrons, the Pauling electronegativity is related to the strength of covalent bonds, which highly influence the SHG coefficients. For example, the substitution of S (PE = 2.58) to Se (PE = 2.55) to Te (PE = 2.10) enhances the SHG coefficients (from AGS (*d*_36_ = 13.4 pm/V), AGSe (*d*_36_ = 33.0 pm/V) to AGTe (*d*_36_ = 99.5 pm/V)). Therefore, we choose the electronegativity of the anion X as the forth feature.

### Performance evaluation

The performance of the RFR model is evaluated through two common quantitative measures, root mean squared error (*RMSE*) and coefficient of determination (*R*^2^)

4.1 $$RMSE=\sqrt{\mathop{\sum }\limits_{i=1}^{m}\,\frac{1}{m}{(f({x}_{i})-{y}_{i})}^{2}}$$

4.2 $${R}^{2}=1-\frac{MSE}{Var(y)}$$

*f*(*x*_*i*_) is the predicted value of the model, *y*_*i*_ is the target variable and *Var*(*y*) is the variance of the sample data. A model with smaller *RMSE* and *R*^2^ closer to 1 will have a higher level of prediction ability.

## Results and Discussion

**Figure 3:**
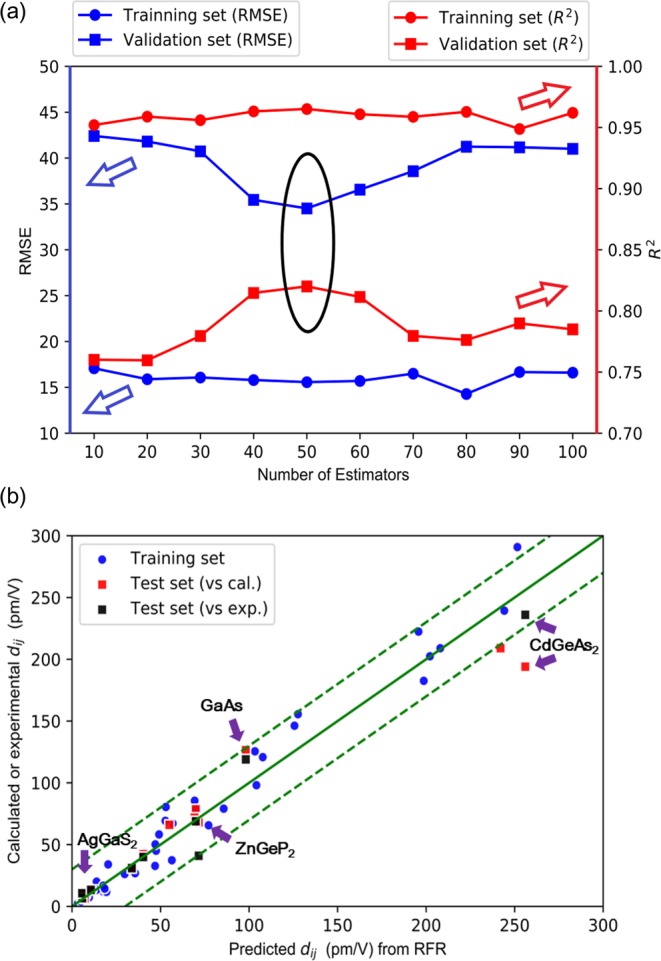
Performance evaluation and model prediction. (**a**) The *RMSE* and *R*^2^ of training and validation set with the change of number of estimators. (**b**) Comparison of DFT training data or experimental data with RFR model predictions for *d*_ij_. Blue circles represent the training and validation data; red squares represent the test data and the y axis is the calculated value; black squares represent the test data and the y axis is the experimental value. The error is less as the point approaching the green line (y = x).

We selected 6 typical NLO crystals (CdSe, GaAs, AgGaS_2_, AgGaSe_2_, ZnGeP_2_, CdGeAs_2_) and 3 new quaternary compounds (Cu_2_CdSnS_4_, Li_2_SrGeS_4_, Li_2_SrSnS_4_) from the screened 61 DL structures as the test set. These crystals have been fully investigated so that the accurate SHG coefficients or credible powder SHG data were obtained. Thus, the RFR model’s results with the first-principles and experimental values can be well compared to get a persuasive evaluation of the model. Meanwhile, another 4 compounds (Cu_3_SbS_4_, Cu_2_CdSnSe_4_, Cu_2_CdSnTe_4_, Li_2_CuPS_4_, also in the 61 DL structures), which don’t have the experimental SHG values but have been investigated computationally, were added. So, the final test set includes 13 NLO crystals (listed in Table 1). The rest 48 crystals in the dataset were randomly split using “bootstrapping” to produce the training and validation set. After repeating 100 random “bootstrapping” and training, we found that the RFR model with the number of estimators 50 yielded a small *RMSE* as well as a high *R*^2^ (indicated in the black circle in Fig. 3a). Then, the test set was input into the trained RFR model with 50 estimators. Figure 3b shows the RFR predicted results and their comparison with first-principles or experimental values.

**Table 1:** Space groups, band gaps and SHG coefficients of DL-type crystals in the test set.

Formula	Space Group	*E*_g_ (eV)	SHG *d*_ij_(pm/V)		
			P. v.^§^	C.v.^§^	E.v.^§^
CdSe	F-43m	1.74	39.18	42.16	40^56^
GaAs	F-43m	1.42	94.73	126.46	119^6^
AgGaS_2_	I-42d	2.64	11.23	16.64	13.4^44^
AgGaSe_2_	I-42d	1.80	73.83	67.99	41.4^44^
ZnGeP_2_	I-42d	2.05	74.03	78.57	68.9^28^
CdGeAs_2_	I-42d	0.57	254.32	194.04/(904.08*)	236^51^
Cu_2_CdSnS_4_	I-42m	1.80	34.46	25.42	31^57^
Li_2_SrGeS_4_	I-42m	3.75	5.48	4.75	0.5*AGS^58^
Li_2_SrSnS_4_	I-42m	3.10	5.76	6.64	0.8*AGS^58^
Cu_3_SbS_4_	I-42m	0.88	56.47	66.06	
Cu_2_CdSnSe_4_	I-42m	0.98	61.68	71.80/(223*)	
Cu_2_CdSnTe_4_	I-42m	0.80	239.05	209.05/(528*)	
Li_2_CuPS_4_	I-4	3.30	7.66	6.40	

^*^First-principles value when upshifting the bands to agree the experimental band gap.

^§^P.v., C.v. and E.v. refer to RFR predicted value, first-principles value and experimental value, respectively.

The results in Fig. 3 and Table 1 demonstrate that the RFR model performs well on the studied DL NLO crystals. The predicted SHG coefficients are in good agreement with the first-principles values, proving that the RFR model has reached within the accuracy of first-principles simulations.

Notably, the RFR model does not need the scissors operator which is vital in the first-principles simulations. When calculating the optical properties of crystals with DFT, the scissors operator is usually used to shift upward all the conduction bands to agree with the experimental band gap^9^. But for those crystals with a small band gap *E*_g_ (<1 eV), this type of scissors sometimes make the first-principles simulation fail to give the accurate optical properties^51^. Another useful method to determine the scissors operator is by aligning the first peak of the calculated conduction bands with the corresponding experimental peak. In Table 1 the first-principles SHG coefficient values using these two kinds of scissors operators and the RFR results for three NLO crystals with small *E*_g_ (CdGeAs_2,_ Cu_2_CdSnSe_4_ and Cu_2_CdSnTe_4_) are listed. CdGeAs_2_ is an important ternary NLO crystal because the experiments demonstrated that it has extremely high SHG coefficient (*d*_36_ = 236 pm/V)^29,51^. The first type of scissors operator gives *d*_36_ = 904.08 pm/V, while the second gives *d*_36_ = 194.04 pm/V. The remaining difference between the calculation and the experiment may come from the fact that the first-principle parameters are selected to balance the cost of time and accuracy. But clearly, the latter first-principles result agrees better with the experimental value, which is also correctly predicted by the RFR model (254.32 pm/V). The similar situations occur in the cases of Cu_2_CdSnSe_4_ and Cu_2_CdSnTe_4_; the comparison of the first-principles results by adopting the first and second types of scissors operators for these two compounds are 223 pm/V *vs*. 71.80 pm/V (Cu_2_CdSnSe_4_) and 528 pm/V *vs*. 209.05 pm/V (Cu_2_CdSnTe_4_), respectively. Considering the fact that the experimental value of Cu_2_CdSnS_4_ is *d*_36_ = 31 pm/V, the choice of the second type of scissors operator is more reasonable. In comparison, the RFR predicted SHG coefficients for Cu_2_CdSnSe_4_ and Cu_2_CdSnTe_4_ are *d*_36_ = 61.68 pm/V and 239.05 pm/V, respectively, which are also very reasonable and independent of the choice of scissors operator. Therefore, the RFR model bypasses the scissors operator problem presented in the first-principles calculations and can obtain the accurate predictions, since it performs on the basic chemical and physical information in crystals.

Moreover, it should be emphasized that our RFR model is successful for the prediction of SHG coefficients in considerable variation of chemical constituents from binary, ternary to quaternary crystals. Thus, this method has the good capability to explore new NLO crystals in the DL system. In particular, two new NLO crystals, Li_2_CuPS_4_ and Cu_2_CdSnTe_4_ with superior mid-IR NLO performances were identified by this model.

Cu_2_CdSnTe_4_ crystallizes in the stannite structure type with the space group I-42m. Dong *et al*.^52^ synthesized this compound by direct reaction of the corresponding elements and discussed its temperature dependent transport properties. In addition, the structural, optoelectronic and thermoelectric properties of Cu_2_CdSnTe_4_ were theoretically studied, which showed that this compound is a potential candidate in the fields of solar cell and thermoelectric^53^. However, the NLO properties of Cu_2_CdSnTe_4_ have not been paid attention. We predict that Cu_2_CdSnTe_4_ is a promising NLO crystal with a band gap 0.8 eV and the SHG coefficient value *d*_36_ = 239.05 pm/V. As a comparison, CdGeAs_2_ owns the currently largest SHG coefficient value (*d*_36_ = 236 pm/V) in known inorganic materials, but its band gap is only 0.57 eV. Therefore, Cu_2_CdSnTe_4_ is expected to have a higher LDT than CdGeAs_2_ in the case of comparable SHG effects. We encourage further experiments to verify our predictions.

Li_2_CuPS_4_ was predicted by Zhu *et al*.^54^ as a sulfide-based super ionic conductor with the kesterite structurel. The higher ionic conductivity arising from the smaller Li ion binding and the reduced electronegativity difference between the anion element and non-lithium cation elements enables Li_2_CuPS_4_ as a promising solid-state electrolyte material. Though this crystal hasn’t been synthesized experimentally, the first-principle calculations have provided the reliable structure information and electronic properties. Its calculated band gap is 3.3 eV using the HSE06 hybrid functional. After inputting the related features of Li_2_CuPS_4_, our model predicts that it has the SHG coefficient 7.66 pm/V (~0.6 × AGS), which can be a potential NLO crystal once experimentally synthesized. Compared to AGS (*E*_g_ = 2.64 eV), Li_2_CuPS_4_ is likely to have the smaller SHG response but the higher LDT, which accords with the inverse relationship between the band gap and the SHG response.

## Conclusion

An HTC and ML workflow has been designed and performed to screen for DL crystals with good balance on the band gap and the SHG coefficient. By selecting four distinctive descriptors, i.e., band gap, electronegativity, group volume and bond flexibility, the predicted results using RFR model are in good agreement with the first-principle calculations, especially on some representative crystals like AgGaS_2_, ZnGeP_2_ and CdGeAs_2_. More interestingly, this fast workflow is independent of the selection of scissors operators, making it more practical. Additionally, this model can be used to facilitate the research of new DL systems. Two unexplored quaternary crystals with good NLO properties, Li_2_CuPS_4_ and Cu_2_CdSnTe_4_, are predicted by this model and wait for the experimental investigations. In summary, this new method opens opportunities for the fast design of NLO crystals with targeting properties.

## Supplementary information

Dataset.
