# Supplementary material for: Data-driven prediction of diamond-like infrared nonlinear optical crystals with targeting performances
Source: Sci Rep. 2020 Feb 26;10:3486. doi: 10.1038/s41598-020-60410-x (PMC7044425; doi:10.1038/s41598-020-60410-x)
Supplement: Supplementary file 1 — Dataset. [file 41598_2020_60410_MOESM1_ESM.docx]

Data-driven prediction of diamond-like infrared nonlinear optical crystals with targeting performances

Rui Wang,^1^ Fei Liang,^1,2^ and Zheshuai Lin^1,2,*^

1 University of Chinese Academy of Sciences, Beijing 100190, China

2 Technical Institute of Physics and Chemistry, Chinese Academy of Sciences, Beijing 100190, China

Correspondence and requests for materials should be addressed to Z.L. (*email: [zslin@mail.ipc.ac.cn](mailto:zslin@mail.ipc.ac.cn))

Table S1. The total 61 DL-type crystals’ formula, space groups, experimental band gaps and calculated SHG coefficients

| Formula | Space Group | *E*_g_ (eV) | SHG *d*_ij_(pm/V) | Ref. |
| --- | --- | --- | --- | --- |
| BeS | F-43m | 5.50 | 1.29 | [1] |
| BeSe | F-43m | 4.30 | 2.18 | [1] |
| BeTe | F-43m | 2.70 | 4.00 | [1] |
| BP | F-43m | 2.10 | 11.81 | [2] |
| BAs | F-43m | 1.86 | 11.78 | [3] |
| MgS | F-43m | 4.80 | 2.96 | [2] |
| MgSe | F-43m | 4.05 | 7.32 | [2] |
| MgTe | F-43m | 3.49 | 20.06 | [2] |
| AlP | F-43m | 2.50 | 13.47 | [2] |
| AlAs | F-43m | 2.23 | 26.89 | [2] |
| AlSb | F-43m | 1.60 | 68.34 | [2] |
| CuCl | F-43m | 3.40 | 4.10 | [2] |
| CuBr | F-43m | 2.91 | 5.27 | [2] |
| CuI | F-43m | 2.95 | 8.45 | [2] |
| ZnS | F-43m | 3.54 | 10.13 | [4] |
| ZnSe | F-43m | 2.68 | 34.01 | [5] |
| ZnTe | F-43m | 2.25 | 67.19 | [6] |
| GaP | F-43m | 2.27 | 45.09 | [7] |
| GaAs | F-43m | 1.42 | 126.46 | [8] |
| CdS | F-43m | 2.34 | 13.89 | [9] |
| CdSe | F-43m | 1.74 | 42.16 | [10] |
| CdTe | F-43m | 1.51 | 125.52 | [11] |
| InP | F-43m | 1.42 | 65.67 | [2] |
| InAs | F-43m | 0.35 | 208.95 | [2] |
| InSb | F-43m | 0.18 | 239.36 | [2] |
| HgS | F-43m | 2.10 | 69.31 | [12] |
| LiGaTe_2_ | I-42d | 2.31 | 43.53 | [13] |
| LiInSe_2_ | I-42d | 2.86 | 13.67 | [13] |
| LiInTe_2_ | I-42d | 1.50 | 79.12 | [14] |
| CuAlSe_2_ | I-42d | 2.67 | 7.23 | [15] |
| CuAlTe_2_ | I-42d | 2.06 | 32.78 | [15] |
| CuGaS_2_ | I-42d | 2.20 | 14.46 | [16] |
| CuGaSe_2_ | I-42d | 1.68 | 80.48 | [15] |
| CuGaTe_2_ | I-42d | 1.12 | 222.42 | [15] |
| CuInS_2_ | I-42d | 1.53 | 26.23 | [17] |
| CuInSe_2_ | I-42d | 1.01 | 158.26 | [17] |
| CuInTe_2_ | I-42d | 0.95 | 182.58 | [17] |
| Cu_3_SbS_4_ | I-42d | 0.88 | 66.06 | [18] |
| ZnSiP_2_ | I-42d | 2.07 | 58.27 | [15] |
| ZnSiAs_2_ | I-42d | 1.74 | 120.81 | [15] |
| ZnGeP_2_ | I-42d | 2.05 | 78.57 | [15] |
| ZnGeAs_2_ | I-42d | 1.15 | 202.45 | [15] |
| ZnSnP_2_ | I-42d | 1.66 | 98.10 | [2] |
| ZnSnAs_2_ | I-42d | 0.63 | 360.46 | [2] |
| AgAlS_2_ | I-42d | 3.13 | 5.11 | [15] |
| AgAlSe_2_ | I-42d | 2.55 | 16.79 | [15] |
| AgAlTe_2_ | I-42d | 2.27 | 50.23 | [15] |
| AgGaS_2_ | I-42d | 2.64 | 16.64 | [15] |
| AgGaSe_2_ | I-42d | 1.80 | 67.99 | [15] |
| AgInSe_2_ | I-42d | 1.24 | 37.38 | [15] |
| AgInTe_2_ | I-42d | 0.95 | 290.84 | [15] |
| CdSiP_2_ | I-42d | 2.20 | 85.53 | [2] |
| CdSiAs_2_ | I-42d | 1.55 | 155.56 | [2] |
| CdGeP_2_ | I-42d | 1.73 | 146.21 | [2] |
| CdGeAs_2_ | I-42d | 0.57 | 194.04 | [2] |
| Li_2_SrGeS_4_ | I-42m | 3.75 | 4.75 | [19] |
| Li_2_SrSnS_4_ | I-42m | 3.10 | 6.64 | [19] |
| Li_2_CuPS_4_ | I-4 | 3.30 | 6.40 | [20] |
| Cu_2_CdSnS_4_ | I-42m | 1.80 | 25.42 | [21] |
| Cu_2_CdSnSe_4_ | I-42m | 0.98 | 71.80 | [21] |
| Cu_2_CdSnTe_4_ | I-42m | 0.80 | 209.05 | [21] |

**References:**

1 Yim, W. M., Stofko, E. J., Paff, R. J. & Dismukes, J. P. Synthesis and some properties of BeTe, BeSe and BeS. *Journal of Physics and Chemistry of Solids* **33**, 501-505, (1972).

2 Madelung, O. *Semiconductors: Data Handbook*. 3 edn, (2004).

3 Hart, G. L. W. & Zunger, A. Electronic structure of BAs and boride III-V alloys. *Phys. Rev. B* **62**, 13522-13537 (2000).

4 Fang, J. *et al.* MOCVD growth of non-epitaxial and epitaxial ZnS thin films. *Applied Surface Science* **70–71**, 701-706 (1993).

5 Kim, Y. *et al.* Bent Polytypic ZnSe and CdSe Nanowires Probed by Photoluminescence. *Small* **13**, 1603695 (2017).

6 Emam-Ismail, M., El-Hagary, M., Shaaban, E. R. & Al-Hedeib, A. M. Microstructure and optical studies of electron beam evaporated ZnSe_1-x_Te_x_ nanocrystalline thin films. *Journal of Alloys & Compounds* **532**, 16-24 (2012).

7 Greil, J. *et al.* Optical Properties of Strained Wurtzite Gallium Phosphide Nanowires. *Nano Letters* **16**, 3703-3709, (2016).

8 Ahtapodov, L. *et al.* A Story Told by a Single Nanowire: Optical Properties of Wurtzite GaAs. *Nano Letters* **12**, 6090-6095, (2012).

9 Cortes, A., Gómez, H., Marotti, R. E., Riveros, G. & Dalchiele, E. A. Grain size dependence of the bandgap in chemical bath deposited CdS thin films. *Solar Energy Materials & Solar Cells* **82**, 21-34 (2008).

10 Ninomiya, S. & Adachi, S. Optical properties of cubic and hexagonal CdSe. *J. Appl. Phys.* **78**, 4681-4689 (1995).

11 Choi, S. B., Man, S. S. & Yong, K. Growth of wurtzite CdTe nanowires on fluorine-doped tin oxide glass substrates and room-temperature bandgap parameter determination. *Nanotechnology* **29** (2018).

12 Mehta, S. K., Kumar, S., Chaudhary, S. & Bhasin, K. K. Nucleation and growth of surfactant-passivated CdS and HgS nanoparticles: Time-dependent absorption and luminescence profiles. *Nanoscale* **2**, 145-152 (2010).

13 Krost, A. *et al.* In situ monitoring of the stress evolution in growing group-III-nitride layers. *Journal of Crystal Growth* **275**, 209-216 (2005).

14 L. Isaenko *et al.* LiGaTe_2_:  A New Highly Nonlinear Chalcopyrite Optical Crystal forthe Mid-IR. *Cryst. Growth Des.* **5**, 1325-1329 (2005).

15 Dey, P. *et al.* Informatics-aided bandgap engineering for solar materials. *Computational Materials Science* **83**, 185-195 (2014).

16 Ullah, S., Mollar, M. & Marí, B. Electrodeposition of CuGaSe_2_ and CuGaS_2_ thin films for photovoltaic applications. *Journal of Solid State Electrochemistry* **20**, 1-7 (2016).

17 Kazmerski, L. L. & Shieh, C. C. Photoconductivity effects in CuInS_2_, CuInSe_2_ and CuInTe_2_ thin films. *Thin Solid Films* **41**, 35-41 (1977).

18 Shi, T., Yin, W. J., Al-Jassim, M. & Yan, Y. Structural, electronic, and optical properties of Cu_3_-V-VI_4_ compound semiconductors. *Applied Physics Letters* **103**, 827 (2013).

19 Wu, K., Chu, Y., Yang, Z. & Pan, S. A_2_SrM^IV^S_4_ (A = Li, Na; M^IV^ = Ge, Sn) Concurrently Exhibiting Wide Bandgaps and Good Nonlinear Optical Responses as New Potential Infrared Nonlinear Optical Materials. *Chemical Science* (2019).

20 Xu, Z. M., Chen, R. H. & Zhu, H. A Li_2_CuPS_4_ superionic conductor: a new sulfide-based solid-state electrolyte. *Journal of Materials Chemistry A* **7**, 12645-12653, (2019).

21 Hussain, S. *et al.* First principles study of structural, optoelectronic and thermoelectric properties of Cu_2_CdSnX_4_ (X=S, Se, Te) chalcogenides. *Materials Research Bulletin* **79**, 73-83 (2016).
